# Comparative Efficacy of Chinese Herbal Injections for the Treatment of Herpangina: A Bayesian Network Meta-Analysis of Randomized Controlled Trials

**DOI:** 10.3389/fphar.2020.00693

**Published:** 2020-05-15

**Authors:** Xiaojiao Duan, Haojia Wang, Jiarui Wu, Wei Zhou, Kaihuan Wang, Xinkui Liu

**Affiliations:** Department of Clinical Chinese Pharmacy, School of Chinese Materia Medica, Beijing University of Chinese Medicine, Beijing, China

**Keywords:** network meta-analysis, Bayesian model, Chinese herbal injections, herpangina, ribavirin

## Abstract

**Background:**

Considering the limitations of broad-spectrum antiviral drugs for the treatment of herpangina and the extensive exploration of Chinese herbal injections (CHIs), systematic evaluation of the efficacy of different CHIs in the treatment of herpangina is a key imperative. In this study, we performed a network meta-analysis to investigate the efficacy of CHIs, including Reduning injection (RDN), Shuanghuanglian injection (SHL), Tanreqing injection (TRQ), Xiyanping injection (XYP), and Yanhuning injection (YHN), in the treatment of herpangina.

**Methods:**

A systematic literature review including studies published before December 17, 2018, was conducted in several databases. The quality of the included studies was assessed using the Cochrane risk of bias tool. Data were analyzed using STATA 13.0 and WinBUGS 1.4.3 software. Surface under the cumulative ranking curve (SUCRA) probability values were applied to rank the examined treatments. Clustering analysis was performed to compare the effects of CHIs between two different outcomes.

**Results:**

A total of 72 eligible randomized controlled trials involving 8,592 patients and five CHIs were included. All patients were under the age of 15 years, and most were under 7 years. The results of the network meta-analysis showed that RDN, XYP, and YHN had significantly better treatment performance than ribavirin. SHL (OR: 0.18; 95% CI: 0.09–0.34) and TRQ (OR: 0.18; 95% CI: 0.10–0.31) were obviously superior to ribavirin with respect to total clinical effectiveness. The results of SUCRA and cluster analysis indicated that RDN is the best intervention with respect to total clinical effectiveness, antipyretic time, and blebs disappearing time. Fifty-four studies described adverse drug reactions/adverse drug events (ADRs/ADEs), and 32 studies reported ADRs/ADEs in detail.

**Conclusions:**

CHIs were found to be superior to ribavirin in terms of treatment performance and may be beneficial for patients with herpangina. RDN had the potential to be the best CHI with respect to all outcome measures. More evidence is needed to assess the safety aspects of CHIs.

## Introduction

Herpangina is a common pediatric disease that is mainly caused by Coxsackie A virus; respiratory and fecal-oral routes are the main routes of transmission. Coxsackie A virus is a small RNA virus that is present in the intestines. The virus exhibits rapid transmission, especially in summer and early autumn. Children in the age group of 1–7 years are particularly vulnerable to infection (Jiang et al., 2015; Huang, 2016). Children infected with herpangina can manifest sore throat, excessive salivation, fever, oral herpes, anorexia, and other symptoms. Enteroviruses are also known to cause serious diseases such as myocardial damage or myocarditis (Wu, 2018; Guo and Li, 2019). Currently, there is no specific treatment for herpangina. Antiviral drugs, symptomatic supportive care, and prevention of complications are the mainstays of treatment (Guo and Li, 2019). Ribavirin is a broad-spectrum antiviral drug that is commonly used for the treatment of herpangina. However, the mechanism of action of ribavirin is highly dependent on viral adenosine kinase; this results in a high probability of the development of drug resistance, which in turn affects the therapeutic effect (Li and Zhan, 2017; Liu et al., 2019). Several recent studies have documented the efficacy of Chinese herbal injections (CHIs) in the treatment of herpangina (Zhu et al., 2014; Xu et al., 2016; Xia, 2016). However, several varieties of CHIs have been used to treat herpangina, and further research is required to identify the best type of CHI for this purpose. Therefore, in this study, we used the network meta-analysis (NMA) method to systematically evaluate the efficacy of different CHIs in the treatment of herpangina. The objective was to identify an optimal intervention measure and provide a basis for clinical drug use.

## Methods

This study is reported in strict accordance with the standard format of the Preferred Reporting Items for Systematic Reviews and Meta-Analysis Specification: PRISMA Extension Statement specification (Hutton et al., 2015; Ge et al., 2017).

### Search Strategy

PubMed, the Cochrane Library, Embase, the Chinese Biological Medicine Literature Service System (SinoMed), the China National Knowledge Infrastructure (CNKI) database, the Chinese Scientific Journal Database (VIP), and the Wanfang Database were searched for randomized controlled trials (RCTs) of CHIs for the treatment of herpangina. Studies published as of December 17, 2018 were eligible for inclusion. In addition, the reference lists of the included studies were manually searched to identify relevant literature. There were three parts of the search strategy, including herpangina, Chinese herbal injection, and random controlled trial. A total of 132 types of CHIs incorporating national standards of the Chinese Food and Drug Administration and 36 kinds of Chinese medicine-derived chemical injections were included in the prescreening. The five CHIs that were finally included in the analysis were Reduning injection (RDN), Shuanghuanglian injection (SHL), Tanreqing injection (TRQ), Xiyanping injection (XYP), and Yanhuning injection (YHN). The detailed search strategy is described in **Presentation File**.

### Inclusion Criteria

#### Types of Studies

RCTs of CHIs for the treatment of herpangina were eligible if they were referred to as “random,” with or without blinding.

#### Types of Participants

All patients included were clinically diagnosed with herpangina according to clear diagnostic criteria, with no limitations of sex, race, or age.

#### Types of Interventions

The interventions included were comparisons between CHIs and ribavirin or between different types of CHIs. Ribavirin and CHIs were administered intravenously; in addition, according to the patient’s condition, certain symptomatic supportive treatments were adopted (e.g., cooling, rehydration, maintenance of water and electrolyte balance, and antibiotic therapy for concurrent bacterial infection). No limitations were imposed with respect to the dosage or treatment course. No other Chinese medicine or remedies were used, such as decoction, proprietary Chinese medicine, acupuncture, or massage.

#### Types of Outcomes

Outcome indicators included total clinical effectiveness, antipyretic time, blebs disappearing time, and adverse reactions (ADRs)/adverse events (ADEs). Total clinical effectiveness = (total number of patients—;number of patients in whom treatment was ineffective)/total number of patients×100%. The evaluation criteria for efficacy were based on the posttreatment recovery of clinical symptoms and signs; ineffective treatment implies deterioration or no change in symptoms and signs after the treatment course.

### Data Extraction and Quality Assessment

All retrieved studies were managed using NoteExpress software. After excluding duplicates, two researchers independently screened the retrieved studies based on the inclusion and exclusion criteria and extracted the data from the included RCTs. The titles and abstracts of retrieved studies were screened to exclude animal studies, literature reviews, and other unrelated articles. Subsequently, studies that met the inclusion criteria were identified, and their full texts were reviewed. A specially designed form (created using Microsoft Excel 2016 software) was used to extract data pertaining to the following information from the included studies: (1) name of first author and the year of publication; (2) basic characteristics of patients: the numbers of patients in the treatment group and the control group, sex distribution, average age or age range, interventions, and treatment details; (3) outcome measures; and (4) study types and main factors affecting the risk of bias. Any disagreement between two researchers during the screening of studies and extraction of data was resolved by consensus or by consulting a third researcher.

Two authors independently assessed the risk of bias in the included studies in accordance with the risk of bias assessment tool recommended in the Cochrane Handbook 5.1 (Higgins and Green, 2010). The following elements were assessed: (1) selection bias associated with random sequence generation; (2) selection bias associated with allocation concealment; (3) performance bias: blinding of participants and personnel; (4) detection bias: blinding of outcome assessment; (5) attrition bias: integrity of outcome data; (6) reporting bias: selective reporting; and (7) bias from other sources. Each element was categorized as “low risk,” “high risk,” or “unclear.” “Low risk” implies that the implementation method is correct or does not affect the result; “high risk” implies that the implementation method is incorrect and affects the measurement of the result; “unclear” means that the information is insufficient, and the risk of bias cannot be judged. Consensus was attained by discussion or involving a third researcher.

### Data Analysis

WinBUGS 1.4.3 software was used to perform NMA, and the Markov chain Monte Carlo method with random-effects model was performed for Bayesian inference. In the WinBUGS software, the number of iterations was set as 200,000, with the first 10,000 iterations used for burn-in to eliminate the impact of the initial value. On NMA, the odds ratio (OR) and 95% confidence intervals (95% CI) were calculated for the binary outcomes; the mean difference (MD) and 95% CI were calculated for continuous outcomes. When the 95% CI for the OR value did not contain 1 and the 95% CI for MD value did not contain 0, the difference between groups was deemed to be statistically significant. Stata 13.0 software was used to map the network of different interventions for each outcome measure, showing the results of the direct and indirect comparison of CHIs. When using the results of WinBUGS software with Stata software, the surface under the cumulative ranking probability (SUCRA) of different CHIs in each outcome index was obtained. The larger the SUCRA and the higher the ranking, the greater the probability that the CHI is the best intervention. A comparison-adjusted funnel plot was used to assess potential publication bias. If points on both sides of the midline in the funnel diagram were symmetric, which meant the correction guideline was at right angles to the midline, it was considered indicative of no significant publication bias. The cluster analysis method was used to comprehensively analyze and compare interventions for two different outcome indicators; then, the optimal injection variety for the two outcome indicators was obtained. The farther from the origin in the cluster map, the better the effect is in these two outcome indicators. If there was a closed loop, the inconsistency test was used to evaluate the consistency of each closed loop, and the inconsistency factors (IFs) and 95% CI were calculated. When the 95% CI contained 0, the consistency was good; otherwise, the closed loop was considered to exhibit significant inconsistency.

## Results

### Search Results

**Figure 1:**
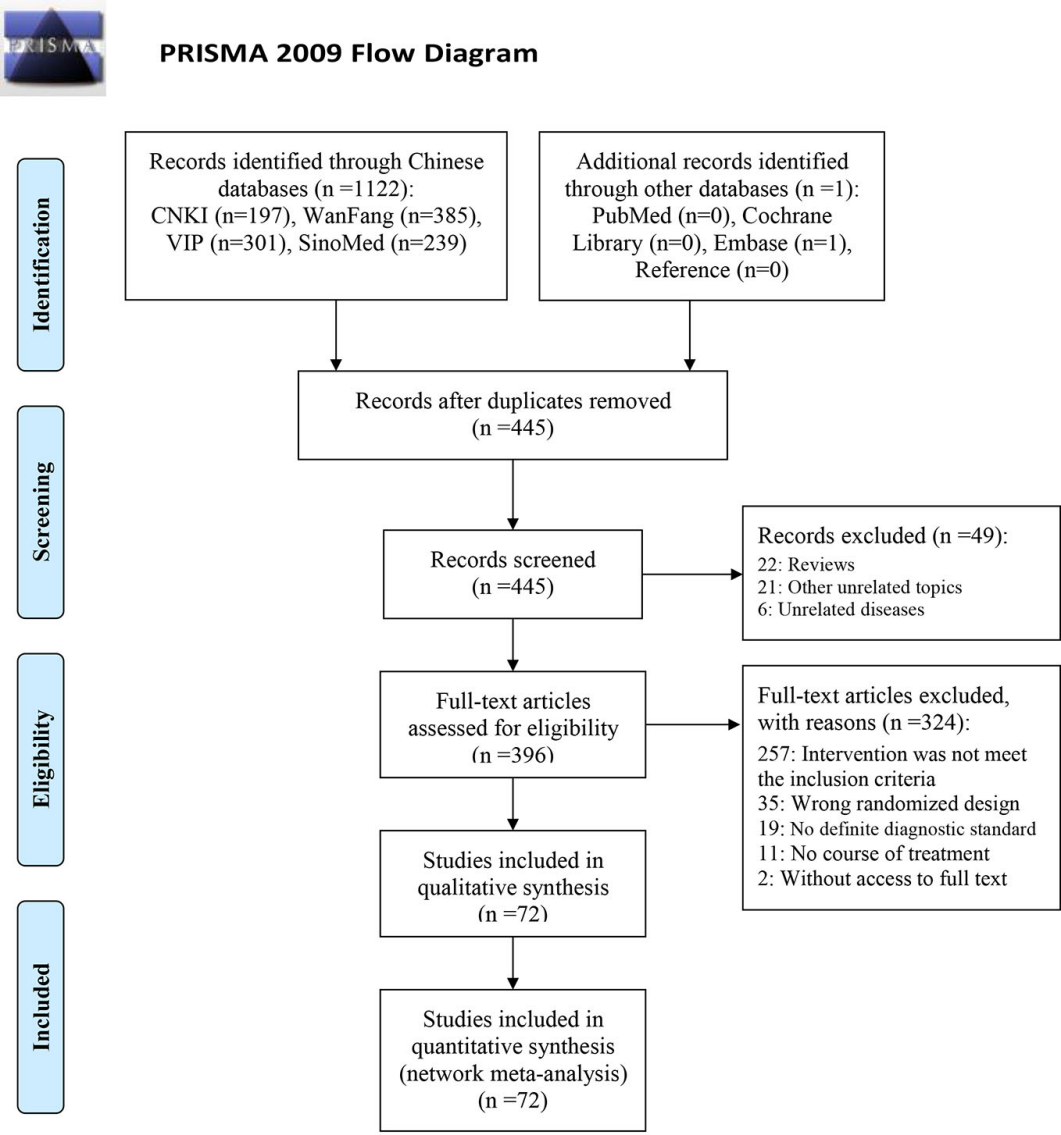
PRISMA flow diagram.

Out of the 1,123 retrieved articles, 72 RCTs (shown in **Table 1**) were selected and included in the NMA. Further details of the literature screening process are presented in **Figure 1**. Two studies were three-arm studies (RDN vs. XYP vs. YHN, and RDN vs. XYP vs. ribavirin), while all other studies were two-arm studies. Among these, 67 RCTs investigated CHIs vs. ribavirin as the intervention, including five kinds of CHIs: RDN (27 RCTs), SHL (4 RCTs), TRQ (5 RCTs), XYP (18 RCTs), and YHN (13 RCTs). The remaining three RCTs investigated CHI vs. another CHI as the intervention: RDN vs. YHN (2 RCTs) and TRQ vs. SHL (1 RCT). All included studies were published in Chinese, and the year of publication ranged from 2007 to 2018.

**Table 1:** Characteristics of the studies included in this meta-analysis.

Study ID	Random method	Cases (A/B/C)	Sex (M/F)	Age	Intervention A	Intervention B	Intervention C	Basic treatment	Course (d)	Consistent baseline	Outcomes	ADRs/ ADEs
Xie, 2017	Random	40/40	47/33	A:3–12(8.8 ± 1.1) B:3–12(8.8 ± 1.3)	RDN: (age) 3–5 < 10 ml; 6–10 = 10 ml; 11–12 = 15 ml	Ribavirin: 10 mg/(kg·d)	NA	NA	5–7	Y	①②③	NR
Xiao, 2016	Random	40/40	43/37	A:3–7 B:3–7	RDN: 0.5–0.8 ml/(kg·d)	Ribavirin: 10 mg/(kg·d)	NA	Rehydration; cooling	5	Y	①	NR
Feng et al., 2015	Random number table	45/45	51/39	A:0.6–7(3.8 ± 2.2) B:0.5–7(3.6 ± 2.3)	RDN: 0.6 ml/(kg·d)	Ribavirin: 10 mg/(kg·d)	NA	NA	5	Y	①②③	N
Liu, 2015	Random	54/51	56/49	0.5–5(3.7 ± 2.2)	RDN: 0.5 ml/(kg·d)	Ribavirin: 10–15 mg/(kg·d)	NA	Symptomatic supportive treatment; bacterial infection combined with antibiotic treatment	3	Y	①②③	N
Wang and Li, 2015	Random	92/90	98/84	A:0.5–5(2.2 ± 1.5) B:0.7–4(2 ± 1.2)	RDN: 0.5 ml/(kg·d)	Ribavirin: 10 mg/(kg·d)	NA	Symptomatic supportive treatment; bacterial infection combined with azithromycin or penicillin treatment	5–7	Y	①②	Detailed description
Deng and Tang, 2014	Random number table	90/90	102/78	0.5–7(3.12 ± 2.22)	RDN: 0.6–0.8 ml/(kg·d)	Ribavirin: 10–15 mg/(kg·d)	NA	Routine care, cooling, rehydration, maintenance of water and electrolyte balance and other symptomatic supportive treatment; bacterial infection combined with antibiotic treatment	5	Y	①②③	Detailed description
Dong, 2014	Random	40/40	45/35	A:0.6–7(3.1 ± 1.2) B:0.5–7(3.4 ± 1.3)	RDN: 0.6 ml/(kg·d)	Ribavirin: 10–15 mg/(kg·d)	NA	Routine care, cooling, rehydration, maintenance of water and electrolyte balance and other symptomatic supportive treatment; bacterial infection combined with antibiotic treatment	3–5	Y	①②③	Detailed description
Hu, 2014	Random	50/50	53/47	0.5–4	RDN: 0.5 ml/(kg·d)	Ribavirin: 10–15mg/(kg·d)	NA	Oral care; antipyretics; vitamin supplements; fluid replacement, etc.	5–7	Y	①②③	Detailed description
Ji et al., 2014	Random number table	95/95	103/87	0–14	RDN: ≤3 (age), 5ml; > 3, 10 ml	Ribavirin: 10–15 mg/(kg·d)	NA	Bacterial infection: plus antiinfection treatment with cephalosporins or penicillin antibiotics; mycoplasma infection: plus macrolide antiinfective treatment, the same symptomatic treatment in both groups	3–5	Y	①②③	NR
Ke, 2014	Random	37/31	37/31	A:1–7(3.5 ± 2.3) B:1–7(3.6 ± 2.1)	RDN: ≤3 (age), 5ml; > 3, 10 ml	Ribavirin: 10–15 mg/(kg·d)	NA	Symptomatic treatment	3	Y	①②③	Detailed description
Tan, 2014	Random number table	110/110	130/90	A:0.4–7(3.6 ± 2.5) B:0.4–7(3.9 ± 2.1)	RDN: 0.5–0.8 ml/(kg·d)	Ribavirin: 10–15 mg/(kg·d)	NA	Routine care, cooling, rehydration to maintain water and electrolyte balance and other symptomatic supportive treatment; bacterial infection plus oral antibiotics	5–7	Y	①②③	Detailed description
Yu and Qian, 2014	Random number table	60/60	76/44	A:0.4–7(3.6 ± 2.5) B:0.4–7(3.8 ± 2.2)	RDN: 0.5–0.8 ml/(kg·d)	Ribavirin: 10 mg/(kg·d)	NA	Routine care, cooling, rehydration to maintain water and electrolyte balance and other symptomatic supportive treatment; bacterial infection plus oral antibiotics	5	Y	①②③	N
Yang, 2013	Random	56/56	60/52	0.8–4	RDN: 0.5–0.7 ml/(kg·d)	Ribavirin: 10 mg/(kg·d)	NA	Routine symptomatic, supportive, antiinfective treatment	5–7	Y	①	N
Zhang, 2013	Random	23/19	25/17	0–14	RDN: ≤3 (age), 5ml; > 3, 10 ml	Ribavirin: 10–15 mg/(kg·d)	NA	Children with bacterial infection use antiinfective treatment with cephalosporin or penicillin, the same symptomatic treatment in both groups	3–5	Y	①②③	Detailed description
Chen, 2012	Random	54/54	NR	1–7	RDN: 0.5–0.8 ml/(kg·d)	Ribavirin: 10 mg/(kg·d)	NA	Symptomatic, support, antiinfective treatment	3–5	Y	①②③	Detailed description
Pu, 2012	Random	50/50	53/47	0.5–6	RDN: 0.6 ml/(kg·d)	Ribavirin: 10–15 mg/(kg·d)	NA	Cooling; antiinfectives with azithromycin or penicillin	3–5	Y	①②③	Detailed description
Wang, 2012	Random	92/76	NR	NR	RDN: 0.6 ml/(kg·d)	Ribavirin: 10–15 mg/(kg·d)	NA	Routine care, cooling, rehydration to maintain the balance of water and electricity and other symptomatic supportive treatment; bacterial or mycoplasma infection plus related antibiotics	3–5	Y	①②③	N
Zhang et al., 2012	Random	96/96	111/81	A:0.8–12(5.2 ± 1.5) B:0.7–7(5.0 ± 1.7)	RDN: 0.5 ml/(kg·d)	Ribavirin: 10 mg/(kg·d)	NA	NA	3	Y	①	Detailed description
Zhang, 2012	Random number table	100/100	113/87	1–7(3.23 ± 2.22)	RDN: 0.5–0.7 ml/(kg·d)	Ribavirin: 10–15 mg/(kg·d)	NA	Routine care, cooling, rehydration to maintain water and electrolyte balance and other symptomatic supportive treatment; bacterial infection plus antibiotic treatment	5	Y	①②③	N
Cai, 2011	Random	60/60	68/52	0.5–7(4.12 ± 3.22)	RDN: 0.6 ml/(kg·d)	Ribavirin: 10–15mg/(kg·d)	NA	Routine care, cooling, rehydration to maintain water and electrolyte balance and other symptomatic supportive treatment; bacterial infection plus antibiotic treatment	3–5	Y	①②③	N
Zeng, 2011	Random	50/50	58/42	1–14 (7.5)	RDN: 0.6 ml/(kg·d)	Ribavirin: 10–15 mg/(kg·d)	NA	NA	5–7	Y	①	NR
Sun et al., 2011	Random	44/44	51/37	0.6–8(3.9 ± 3.2)	RDN: 0.5–0.8 ml/(kg·d)	Ribavirin: 10 mg/(kg·d)	NA	Symptomatic supportive treatment; bacterial infection plus antibiotic treatment	5–7	Y	①②③	Detailed description
Xie, 2011	Random	45/45	48/42	0.5–6	RDN: 0.6 ml/(kg·d)	Ribavirin: 10–15 mg/(kg·d)	NA	Cooling; bacterial infections with azithromycin or penicillin against infection	3–5	Y	①②③	Detailed description
Guo, 2010	Random	60/60	62/58	0.5–5	RDN: < 2 (age) 0.5–0.8 ml/d	Ribavirin: 10–15mg/(kg·d)	NA	NA	5–7	Y	①	N
Xiao, 2010	Random	53/52	55/50	0.5–7	RDN: 0.5–0.8 ml/(kg·d)	Ribavirin: 10 mg/(kg·d)	NA	Symptomatic supportive treatment; bacterial infection plus antibiotic treatment	5–7	Y	①②③	Detailed description
Xu et al., 2009	Random number table	60/60	64/56	1–7	RDN: 0.6–0.8 ml/(kg·d)	Ribavirin: 10 mg/(kg·d)	NA	NA	3–5	Y	①②③	Detailed description
Pang et al., 2008	Random number table	42/42	53/31	1–7	RDN: 0.6–0.8 ml/(kg·d)	Ribavirin: 10 mg/(kg·d)	NA	Conventional fluid therapy and symptomatic treatment	3–5	Y	①②③	Detailed description
Wang, 2013	Random	60/60	64/56	0.42–5	SHL: 60 mg/(kg·d)	Ribavirin: 10 mg/(kg·d)	NA	Cooling; bacterial infection combined with antibiotic treatment	3–6	Y	①	NR
Zhao, 2012	Random	44/44	54/34	0.58–5	SHL: 60 mg/(kg·d)	Ribavirin	NA	Basic oral care; oral multivitamin B	3	NR	①	N
Peng and Tao, 2010	Random	66/40	63/43	0–14	SHL: 60 mg/(kg·d)	Ribavirin: 10–15 mg/(kg·d)	NA	Oral care; bacterial infection combined with antibiotic treatment	3–7	Y	①	N
Cao, 2008	Random	40/36	46/30	0.67–5	SHL: 60 mg/(kg·d)	Ribavirin: 10 mg/(kg·d)	NA	Drink more water; supplement vitamin B, vitamin B family	7	Y	①②	Detailed description
Feng and Peng, 2013	Random	80/72	79/73	A: 0.92 ± 0.5 B: 1 ± 0.42	TRQ: 0.3–0.5 ml/(kg·d)	Ribavirin: 10–15 mg/(kg·d)	NA	Oral care, fluid replacement, symptomatic and other conventional comprehensive treatment	5–7	Y	①	N
Cai, 2012	Random	108/102	110/100	A: 0.42–5.5 B: 0.42–6	TRQ: 0.5 ml/(kg·d)	Ribavirin: 10 mg/(kg·d)	NA	Supplemented with intravenous infusion of water-soluble vitamins; correct water and electrolyte disorders according to the situation; infected patients were given intravenous infusion of cefotiam	5	Y	①	NR
Wen, 2012	Random	24/23	25/22	NR	TRQ: 0.3–0.5 ml/(kg·d)	Ribavirin: 10–15 mg/(kg·d)	NA	NA	3–5	Y	①	N
Tan, 2011	Random	68/62	69/61	A:0.33–10(3.1 ± 2.6)B:0.42–11(2.8 ± 3.3)	TRQ: 0.5 ml/(kg·d)	Ribavirin: 10 mg/(kg·d)	NA	Symptomatic support and other treatments; bacterial infections given antibiotic treatment	5	Y	①②	NR
Jiang, 2009	Random sampling	50/50	54/46	A: 1 ± 0.42 B: 1.1 ± 0.33	TRQ: 0.3–0.5 ml/(kg·d)	Ribavirin: 0.1 mg/(kg·d)	NA	NA	3	Y	①	NR
Xia, 2016	Random number table	46/40	44/42	1–5	XYP: 0.2 ml/(kg·d)	Ribavirin: 10 mg/(kg·d)	NA	Antipyretic	3	Y	①②③	N
Cao, 2015	Random	25/23	24/24	A:0.7–2.5 B:1–3.2	XYP: 0.2 ml/(kg·d)	Ribavirin: 10 ml/(kg·d)	NA	Antipyretic	5	Y	①	NR
Lin, 2014	Random	48/48	51/45	0.5–3	XYP: 5 mg/(kg·d)	Ribavirin: 10 mg/(kg·d)	NA	Dietary guidance; according to the nature and degree of dehydration, rehydration to correct water, electrolyte and acid-base balance disorders; antipyretic, symptomatic treatment	3	Y	①	N
Yang et al., 2013	Random	123/123	130/116	0.4–6	XYP: 0.2–0.4 ml/(kg·d)	Ribavirin: 15 mg/(kg·d)	NA	Symptomatic supportive treatment	3	Y	①	N
Zeng et al., 2013	Random	60/60	68/52	0.7–5	XYP: 5–10 mg/(kg·d)	Ribavirin: 10 mg/(kg·d)	NA	Cooling; treatment of bacterial infection with cefotaxime	5	Y	①②③	Detailed description
Wang, 2013	Random	45/45	49/41	A:0.7–7 B:0.7–6	XYP: 20 mg/(kg·d)	Ribavirin: 10 mg/(kg·d)	NA	General care; symptomatic, supportive care; multivitamin supplementation	5–7	Y	①②③	NR
Zhou, 2013	Random	72/68	82/58	0.5–5	XYP: 0.2–0.4 ml/(kg·d)	Ribavirin: 10–15 mg/(kg·d)	NA	Symptomatic supportive treatment	5	Y	①②	Detailed description
Su and Ke, 2012	Random number table	195/194	202/187	1–7	XYP: 0.1–0.2 ml/(kg·d)	Ribavirin: 10 mg/(kg·d)	NA	Routine rehydration and symptomatic treatment, if the child’s temperature is >38.50°C, use short-acting antipyretic agent as appropriate	3–5	Y	①②③	Detailed description
Jia and Tian, 2012	Random	70/50	76/44	0.5–5	XYP: 5 mg/(kg·d)	Ribavirin: 10–15 mg/(kg·d)	NA	Symptomatic supportive treatment	5	Y	①	Detailed description
Li et al., 2011	Random	39/37	42/34	A:0.3–7 B:0.25–7	XYP: 0.2–0.4 ml/(kg·d)	Ribavirin: 10–15 mg/(kg·d)	NA	Basic treatment of respiratory tract isolation, symptomatic treatment, supportive treatment, etc.	3	Y	②③	N
Yang, 2011	Random	31/30	NR	0.3–5	XYP: 10 mg/(kg·d)	Ribavirin: 10 mg/(kg·d)	NA	Symptomatic treatment such as fever, vitamin B supplements and fluid replacement	5	Y	①	NR
Zhang, 2011	Random sampling	34/38	26/46	A:(1.3 ± 0.41) B:(1.5 ± 0.43)	XYP: 5–8 mg/(kg·d)	Ribavirin: 10–15 mg/(kg·d)	NA	Children with moderate to high fever are given oral or intramuscular injection of antipyretics to cool down	3	Y	①②③	N
Zhang, 2011	Random	42/40	NR	0.3–5	XYP: 10 mg/(kg·d)	Ribavirin: 10 mg/(kg·d)	NA	Symptomatic supportive treatment	5	Y	①②③	Detailed description
He and Peng, 2010	Random	42/38	45/35	A:0.5–4 B:0.5–5	XYP: 5 mg/(kg·d)	Ribavirin: 10 mg/(kg·d)	NA	Symptomatic supportive treatment	5	Y	①②	Detailed description
Shen, 2010	Random	25/25	27/23	A:0.3–3.5 B:0.42–4	XYP: 0.2–0.3 ml/(kg·d)	Ribavirin: 10 mg/(kg·d)	NA	Antipyretic; oral care; secondary bacterial infection plus penicillin or cephalosporin treatment	3	Y	①	N
Guo, 2009	Random	80/80	85/75	1–7	XYP: 5–10 mg/(kg·d)	Ribavirin: 10–15 mg/(kg·d)	NA	Symptomatic supportive treatment	3–5	Y	①	Detailed description
Chen et al., 2008	Random number table	36/33	38/31	1–7	XYP: 0.2–0.4 ml/(kg·d)	Ribavirin: 10 mg/(kg·d)	NA	Routine rehydration and symptomatic treatment, if the child’s temperature is >38.50°C, use short-acting antipyretic agent as appropriate	3	Y	①②③	N
Huang et al., 2008	Random	68/62	76/54	A:0.5–4 B:0.5–5	XYP: 5 mg/(kg·d)	Ribavirin: 10 mg/(kg·d)	NA	Antipyretic; supplemented with vitamin B, vitamin B, ceftriaxone sodium or amoxicillin clavulanate potassium for antiinfective treatment	5	Y	①②	Detailed description
Qu et al., 2016	Random	40/40	45/35	0.5-5	YHN: 5–10 mg/(kg·d)	Ribavirin: 10-15mg/(kg·d)	NA	Give appropriate and supportive care as appropriate	3-5	Y	①	NR
Yang, 2014	Random number table	175/175	189/161	1-7	YHN: 5–10 mg/(kg·d)	Ribavirin: 10 mg/(kg·d)	NA	Give intravenous rehydration and symptomatic treatment, and give ibuprofen antipyretic as appropriate for body temperature >38.5°C	3-5	Y	①②③	Detailed description
Dong and Feng, 2013	Random	40/40	42/38	0.5–7	YHN: 5 mg/(kg·d)	Ribavirin: 10 mg/(kg·d)	NA	High fever given antipyretics; rest; drinking more water; prevention of complications; antibiotics in patients with bacterial infections	5	Y	①	NR
Song and Fan, 2013	Random	40/36	49/27	0.5–2	YHN: 5–10 mg/(kg·d)	Ribavirin: 10–15mg/(kg·d)	NA	Rehydration and symptomatic treatment; bacterial infections treated with antibiotics	5–7	Y	①②③	NR
Li, 2012	Random	42/38	42/38	A:5.6(1–7) B:5.8(1–7)	YHN: 5–10 mg/(kg·d)	Ribavirin: 10–15 mg/(kg·d)	NA	Oral care topical treatment	5	Y	①	Detailed description
Wang et al., 2012	Random	120/120	100/140	0–7	YHN: 3–8 mg/(kg·d)	Ribavirin: 10–15 mg/(kg·d)	NA	NA	3–5	Y	①	NR
Fang, 2011	Random	67/66	69/64	1–7	YHN: 5–10 mg/(kg·d)	Ribavirin: 10 mg/(kg·d)	NA	Conventional fluid replacement and symptomatic treatment; if the body temperature is >38.5°C, use a short-acting antipyretic agent as appropriate.	3	Y	①	N
Guo, 2011	Random	44/44	48/40	0.5/4	YHN: 5–10 mg/(kg·d)	Ribavirin: 10 mg/(kg·d)	NA	Antipyretic; supplemented with vitamin B, vitamin B2, ceftazidime, or cefuroxime for antiinfective treatment	5	Y	①③	Detailed description
Li et al., 2011	Random	40/40	45/35	0.5–5	YHN: 5–10 mg/(kg·d)	Ribavirin: 10–15mg/(kg·d)	NA	Give appropriate and supportive care as appropriate	3–5	Y	①	NR
Yin, 2011	Random	30/30	30/30	A:1–7 B:1.5–6.5	YHN: 5–10 mg/(kg·d)	Ribavirin: 10-15mg/(kg·d)	NA	Give appropriate and supportive care as appropriate	3–5	Y	①②③	NR
Lv, 2009	Random	30/18	28/20	A:0.5–4 B:0.5–3.5	YHN: 5–10 mg/(kg·d)	Ribavirin: 10–5mg/(kg·d)	NA	Pay attention to rest; drink plenty of water; add vitamin B, vitamin B; cool down	5	Y	①②	Detailed description
Hu, 2008	Random	30/30	28/32	0.7–4	YHN: 5–10 mg/(kg·d)	Ribavirin: 10–15mg/(kg·d)	NA	Give appropriate and supportive care as appropriate	4–7	Y	②③	N
Wei, 2007	Random	63/63	NR	1–7	YHN: 5–10 mg/(kg·d)	Ribavirin: 10–15 mg/(kg·d)	NA	Give appropriate and supportive care as appropriate	3	Y	②③	N
Guo et al., 2014	Random	38/35	38/35	A:2.38 ± 1.56 B:2.58 ± 1.54	YHN: 5–10 mg/(kg·d)	RDN: 0.5–0.7 ml/(kg·d)	NA	Intravenous infusion of water-soluble vitamins; oral care; symptomatic treatment; hyperthermia preheat treatment; supplementation of liquids and electrolytes	5–7	Y	①②③	Detailed description
Zhu, 2013	Random	60/60	67/53	1–5	TRQ: 0.5–0.3 ml/(kg·d)	SHL: 60 mg/(kg·d)	NA	Rehydration and symptomatic treatment; infected with antibiotics	NR	Y	①②	NR
Wang, 2012	Random	40/40/40	NR	3.16 ± 2.22	XYP: 5 mg/(kg·d)	RDN: 0.5–0.8 ml/(kg·d)	YHN: 3–5 mg/(kg·d)	All patients were given routine support, cooling, rehydration to maintain water and electrolyte balance and other symptomatic supportive treatment; patients with concurrent bacterial infections were treated with antibiotics	5	Y	①②③	Detailed description
Zhou, 2012	Random	60/60	62/58	0.3–6	RDN: 0.6 ml/(kg·d)	YHN: 10 mg/(kg·d)	NA	Light diet; oral care; those with high fever to physical cooling and antipyretic cooling; those with vomiting and diarrhea to microecological regulators and intestinal mucosal protective agents, supplements with liquids and electrolytes; those with bacterial infections apply appropriate antibiotics, etc.	3–5	Y	①	Detailed description
Liu and Li, 2011	Random	90/90/90	147/123	A:.05–6.5 B:0.5–6.7 C:0.4–6.6	XYP: 5 mg/(kg·d)	Ribavirin: 10 mg/(kg·d)	RDN: 0.5 ml/(kg·d)	Symptomatic support treatment such as antipyretic and drinking water	7	Y	①②③	Detailed description

M, male; F, female; RDN, Reduning injection; SHL, Shuanghuanglian injection; TRQ, Tanreqing injection; XYP, Xiyanping injection; YHN, Yanhuning injection; NA, Not Applicable; NR, Not Reported; Y, Yes; N, No; ①: The Rate of Clinical Efficacy; ②: Antipyretic time; ③: Blebs disappearance time.

### Inclusion Studies and Characteristics

**Figure 2:**
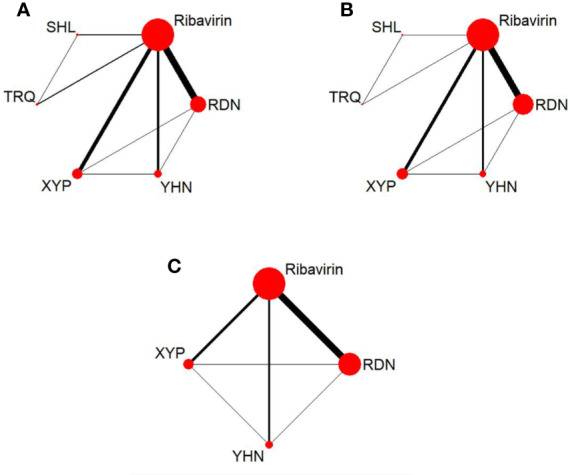
Network graph for different outcomes. **(A)** Total clinical effectiveness; **(B)** antipyretic time; **(C)** Blebs disappearing time. RDN, Reduning injection; SHL, Shuanghuanglian injection; TRQ, Tanreqing injection; XYP, Xiyanping injection; YHN, Yanhuning injetion.

The 72 RCTs included 8,592 patients; of these, 1,866 patients were treated with RDN, 270 patients received SHL, 390 patients received TRQ, 1,211 patients received XYP, 896 patients received YHN, and 3,959 patients received ribavirin. Six studies did not report the sex distribution in the study population; the remaining studies enrolled 4,320 male patients, which accounted for 54.50% (4,320/7,927). All included patients were under the age of 15 years, and most were under 7 years. The maximum sample size of the included RCTs was 195, and the minimum sample size was 18. Sixty-nine RCTs (95.83%, five CHIs) reported total clinical effectiveness, 45 RCTs (62.50%, five CHIs) reported antipyretic time, and 38 RCTs (52.78%, three CHIs) reported blebs disappearing time. The network graph of CHIs with different outcomes is shown in **Figure 2**. All treatment courses lasted < 7 days. The details of the included studies are shown in **Table 1**.

### Methodological Quality

**Figure 3:**
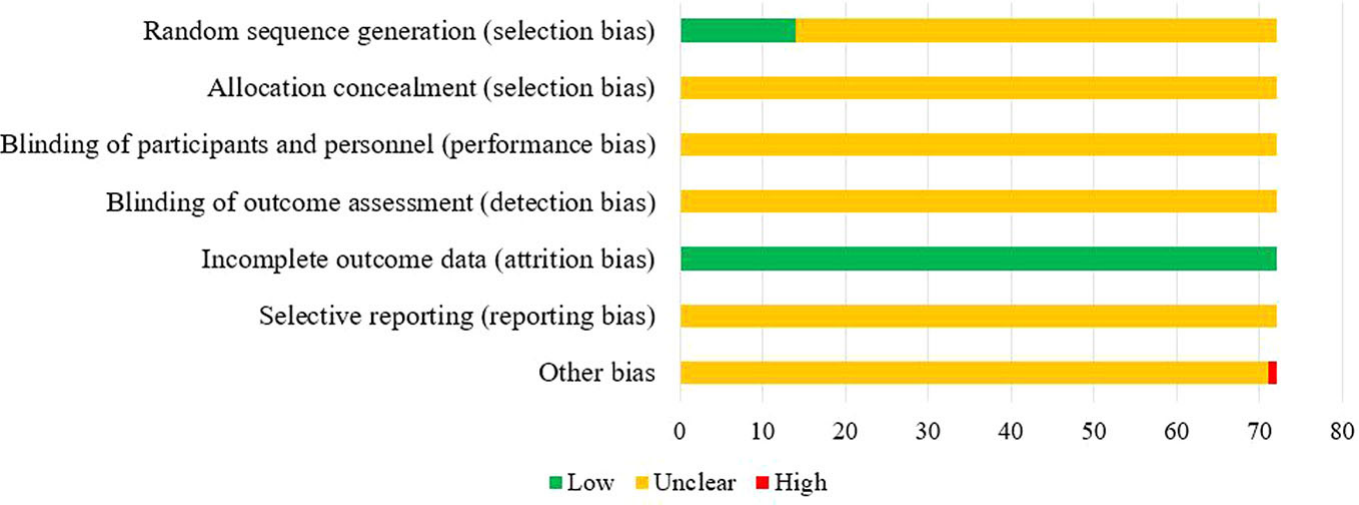
Assessment of risk of bias.

Of the 72 included studies, 12 RCTs used a random number table for group allocation, while two RCTs used a random sampling method. The selection bias associated with random sequence generation of the above studies was evaluated as “low risk.” All studies reported complete data, and their attrition bias was evaluated as “low risk.” One RCT did not indicate whether the baseline characteristics of the two groups were comparable at the time of grouping, which may have impacted the results, and other corresponding biases were evaluated as “high risk.” The risk of bias entries for the remaining studies was rated as “unclear” due to insufficient information. The results of the risk of bias evaluation are shown in **Figure 3**.

### Network Meta-Analysis

#### Total Clinical Effectiveness

Sixty-nine RCTs reported the total clinical effectiveness, involving five CHIs and six interventions. The network graph is shown in **Figure 2**. The OR value of the NMA is shown in **Table 2**. Compared with ribavirin treatment, RDN, SHL, TRQ, XYP, and YHN were found to have greater total clinical effectiveness in patients with herpangina; the between-group differences were statistically significant. There were no significant differences between the remaining intervention groups.

**Table 2:** Statistical results of network meta-analysis for the outcomes [odds ratio (OR)/mean difference (MD) value, 95% CI].

	Clinical total efficiency*	Antipyretic time	Blebs disappearance time
**RDN vs**.			
SHL	1.02 (0.51,2.08)	−0.27 (−3.73,2.70)	–
TRQ	1.00 (0.54,1.84)	−0.34 (−3.56,2.76)	–
XYP	0.75 (0.51,1.13)	−0.26 (−1.06,0.58)	−0.09 (−1.03,0.81)
YHN	0.80 (0.50,1.28)	−0.50 (−1.39,0.41)	−0.42 (−1.44,0.64)
Ribavirin	**0.18 (0.14,0.23)**	**−1.33 (−1.82,-0.80)**	**−1.49 (−1.92,−1.06)**
**SHL vs**.			
TRQ	0.98 (0.47,2.04)	−0.02 (−3.77,3.51)	–
XYP	0.73 (0.36,1.53)	0.05 (−3.00,3.41)	–
YHN	0.79 (0.36,1.69)	−0.17 (−3.28,3.17)	–
Ribavirin	**0.18 (0.09,0.34)**	−1.00 (−3.98,2.33)	–
**TRQ vs**.			
XYP	0.75 (0.39,1.42)	0.08 (−3.04,3.35)	–
YHN	0.80 (0.39,1.59)	−0.14 (−3.34,3.08)	–
Ribavirin	**0.18 (0.10,0.31)**	−0.98 (−4.04,2.21)	–
**XYP vs**.			
YHN	1.07 (0.63,1.79)	−0.25 (−1.21,0.76)	−0.33 (−1.62,0.99)
Ribavirin	**0.24 (0.17,0.33)**	**−1.07 (−1.73,−0.42)**	**−1.40 (−2.24,−0.56)**
**YHN vs**.			
Ribavirin	**0.23 (0.15,0.35)**	**−0.82 (−1.61,−0.08)**	**−1.08 (−2.04,−0.12)**

*indicates that the result is OR; Bold results indicate statistically significant differences between groups; RDN, Reduning injection; SHL, Shuanghuanglian injection; TRQ, Tanreqing injection; XYP, Xiyanping injection; YHN, Yanhuning injection

**Figure 4:**
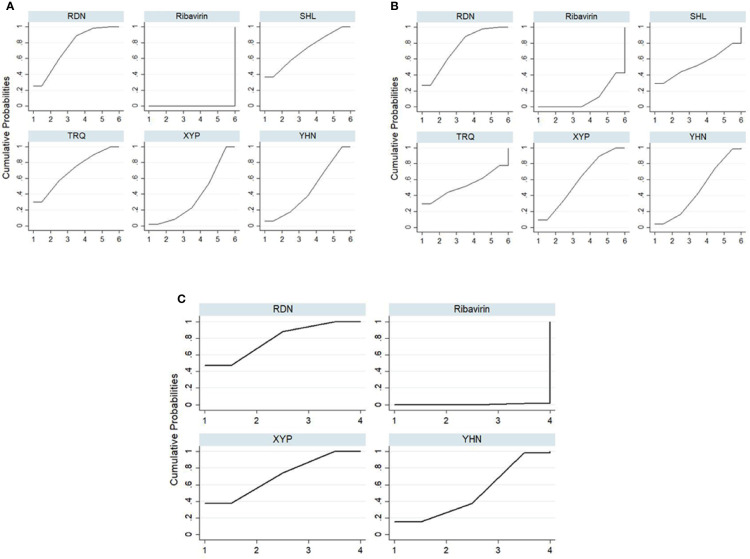
Plot of the surface under the cumulative ranking curves for all treatments. **(A)** Total clinical effectiveness; **(B)** antipyretic time; **(C)** Blebs disappearing time. RDN, Reduning injection; SHL, Shuanghuanglian injection; TRQ, Tanreqing injection; XYP, Xiyanping injection; YHN, Yanhuning injection.

The SUCRA ordering and probability value results (**Figure 4**, **Table 3**) indicate that RDN is the most likely to improve total clinical effectiveness in herpangina patients compared with ribavirin, followed by SHL and TRQ.

**Table 3:** Surface under the cumulative ranking probabilities (SUCRA) results of three outcomes.

Interventions	RDN	SHL	TRQ	XYP	YHN	Ribavirin
Total clinical effectiveness	74.5%	71.3%	70.6%	37.4%	46.2%	0%
Antipyretic time	74.9%	53.9%	53.1%	59.6%	47.5%	11.1%
Blebs disappearance time	78.6%	–	–	70.5%	50.5%	0.5%

The warmer the color, the greater the SUCRA, and the greater the probability of becoming the best intervention.

#### Antipyretic Time

Forty-five RCTs reported antipyretic time, involving five kinds of CHIs and six interventions. The network diagram is shown in **Figure 2**. The results of NMA (**Table 2**) showed that RDN, XYP, and YHN can shorten the antipyretic time compared with ribavirin; between-group differences in this respect were statistically significant. The difference between the remaining interventions was not statistically significant. The SUCRA ordering and probability value results (**Figure 4**, **Table 3**) indicated that RDN has the best treatment effect, followed by XYP and SHL.

#### Blebs Disappearing Time

Thirty-eight RCTs reported the blebs disappearing time; these involved four interventions (RDN, XYP, YHN, and ribavirin). The network diagram is shown in **Figure 2**. On NMA (**Table 2**), RDN, XYP, and YHN were found to be associated with a shorter blebs disappearing time compared with ribavirin; the between-group difference in this respect was statistically significant. No significant between-group differences were observed for other interventions. The SUCRA ordering and probability value results (**Figure 4**, **Table 3**) indicated that RDN has the best treatment effect, followed by XYP and YHN.

### Cluster Analysis

**Figure 5:**
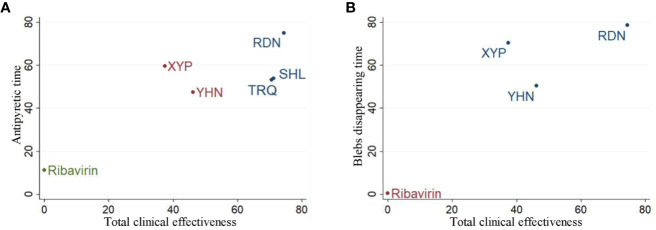
Cluster analysis plot for three outcomes. **(A)** Cluster analysis plot of total clinical effectiveness and antipyretic time; **(B)** cluster analysis plot of Total clinical effectiveness and blebs disappearing time. Interventions with the same color belonged to the same cluster, and interventions located in the upper right corner indicate optimal therapy for two different outcomes; RDN, Reduning injection; SHL, Shuanghuanglian injection; TRQ, Tanreqing injection; XYP, Xiyanping injection; YHN, Yanhuning injection.

The cluster analysis method allowed for a comprehensive comparison of the effects of different interventions on total clinical effectiveness, antipyretic time, and blebs disappearing time. The results showed (**Figure 5**) that RDN was the best intervention in terms of total clinical effectiveness and antipyretic time, total clinical effectiveness and blebs disappearing time; these findings suggest that the efficacy of RDN in the treatment of herpangina is worthy of attention.

### Publication Bias

**Figure 6:**
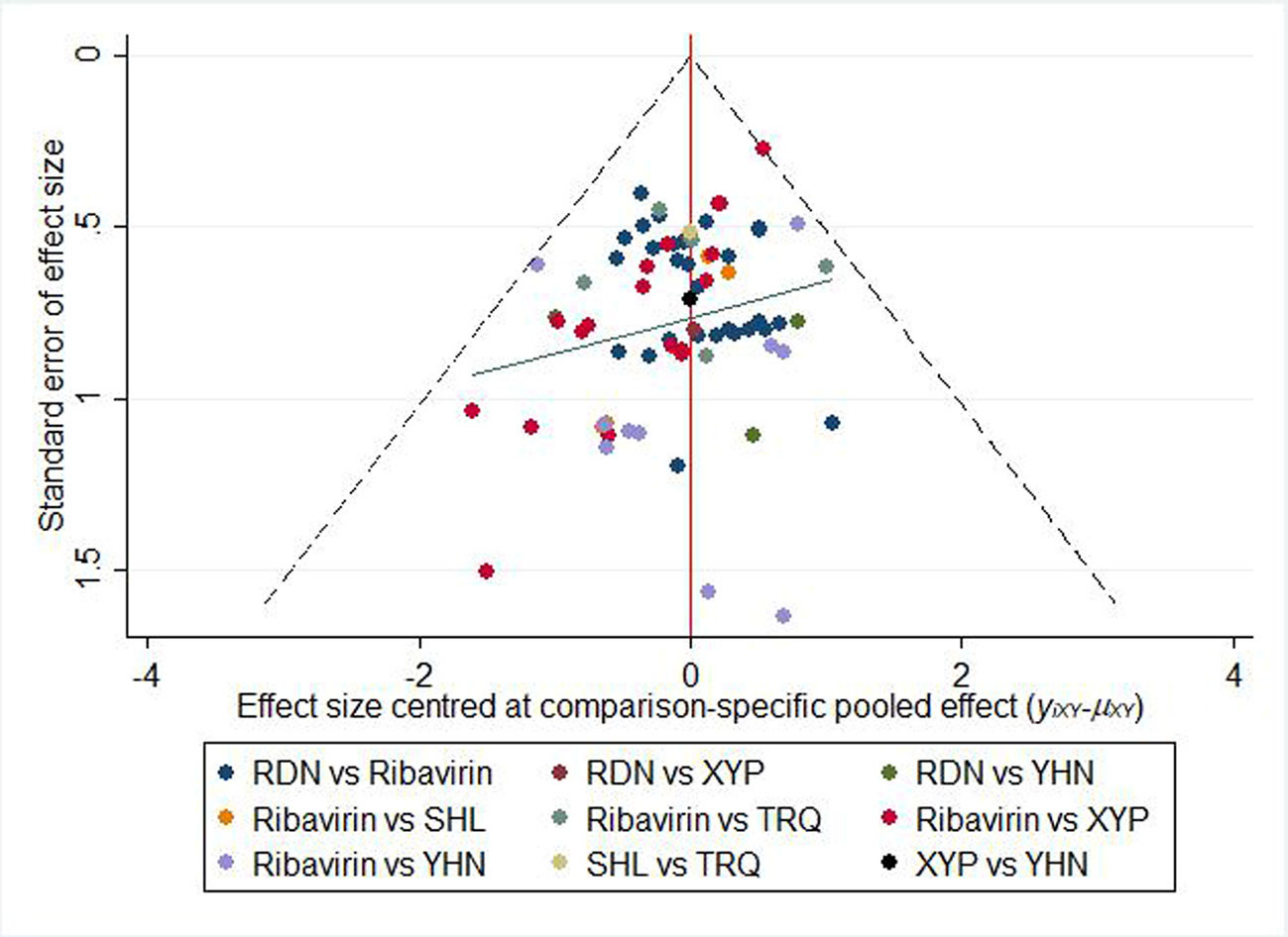
Funnel plot of the clinical effectiveness. RDN, Reduning injection; SHL, Shuanghuanglian injection; TRQ, Tanreqing injection; XYP, Xiyanping injection; YHN, Yanhuning injection.

**Figure 6** shows the comparison-correction funnel plot for total clinical effectiveness to assess potential publication bias. The points on both sides of the centerline of the funnel plot are not completely symmetrical, and there is a large angle between the correction guideline and the centerline. This suggests that our results may have been affected by publication bias to some extent.

### Consistency Test

**Figure 7:**
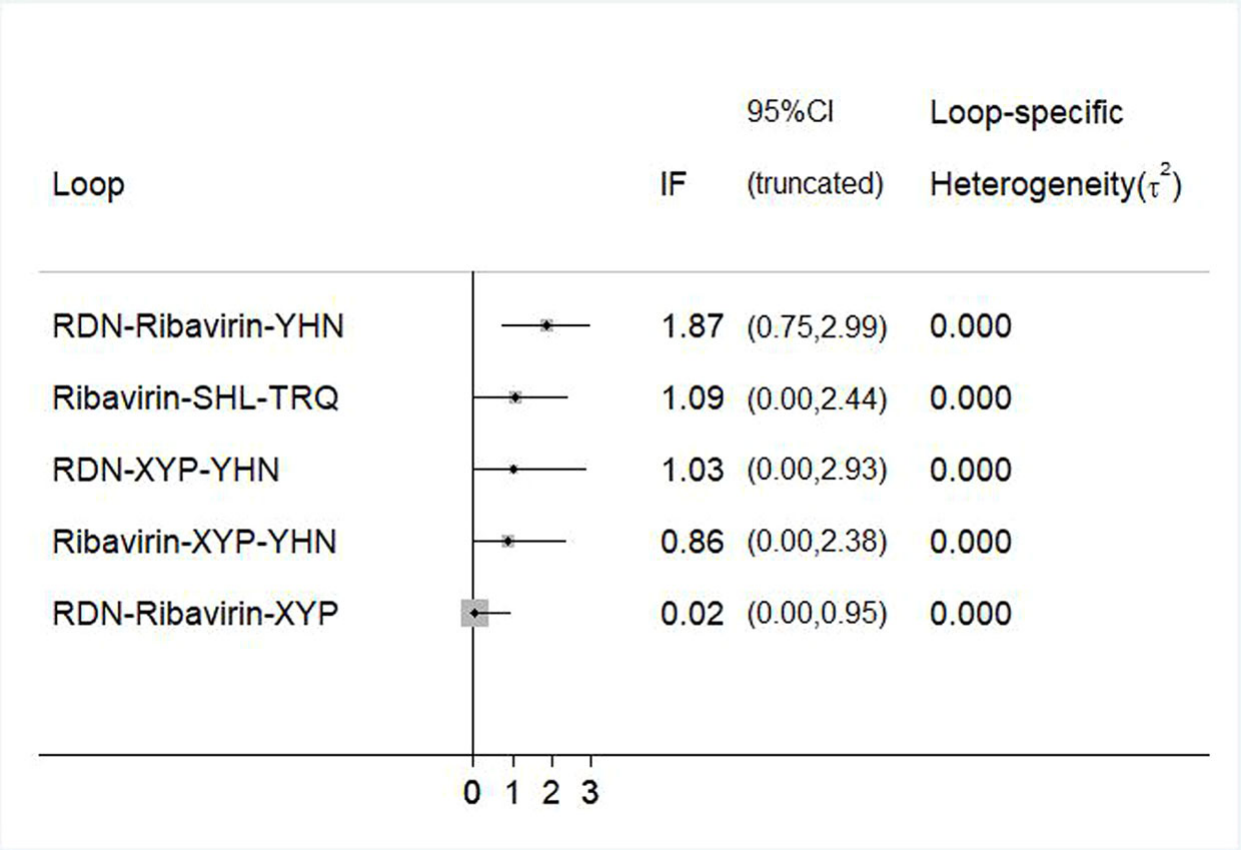
Inconsistency test for the clinical effectiveness. RDN, Reduning injection; SHL, Shuanghuanglian injection; TRQ, Tanreqing injection; XYP, Xiyanping injection; YHN, Yanhuning injection.

To evaluate the consistency of each closed loop, the IF and its 95% CI were calculated using Stata software. When the 95% CI contained 0, it was considered to be consistent; otherwise, there was a significant inconsistency in the closed loop. For example, an inconsistency plot of total clinical effectiveness is shown in **Figure 7**. The inconsistency test results showed the inclusion of five rings, and only the 95% CI of 1 ring did not contain 0; this indicates that there was a small inconsistency in the included studies and that the results were relatively reliable.

### Adverse Drug Reactions/Adverse Drug Events

Of the 72 included studies, 18 (25.00%) did not monitor ADRs/ADEs during treatment. Out of the 54 (75.00%) studies that described ADRs/ADEs, 22 studies recorded no ADRs/ADEs, while 32 studies reported the occurrence and the number of affected patients in detail. The total number of patients who experienced ADRs/ADEs was 6,647, which accounted for 77.36% of the total patients. No ADRs/ADEs on TRQ were reported in the currently included studies; ADRs/ADEs of other interventions are shown in **Table 4**.

**Table 4:** Details of adverse drug reactions (ADRs)/adverse drug events (ADEs).

	Reduning injection	Shuanghuanglian injection	Xiyanping injection	Yanhuning injection	Ribavirin	Total number of cases
Gastrointestinal reaction	1.65% (27/1,641)	1.33% (2/150)	0.72% (8/1,110)	1.71% (10/586)	0.33% (10/3,056)	57
Rash	0.24% (4/1,641)	1.33% (2/150)	0.81% (9/1,110)	1.71% (10/586)	0.65% (20/3,056)	45
Facial flushing			0.18% (2/1,110)	0.34% (2/586)		4
Gastrointestinal reaction with Rash			0.18% (2/1,110)		0.23% (7/3,056)	9
Leukopenia	0.06% (1/1,641)				1.24% (38/3,056)	39
Increased white blood cell count					0.46% (14/3,056)	14
Anemia					0.07% (2/3,056)	2
Breathing suffering, mild chest pain					0.03% (1/3,056)	1
Total	1.95% (32/1,641)	2.67% (4/150)	1.89% (21/1,110)	3.75% (22/586)	3.01% (92/3,056)	171

## Discussion

In this study, we evaluated the use of five types of commonly used CHIs (RDN, SHL, TRQ, XYP, YHN) and ribavirin for the treatment of herpangina. The efficacy of the CHIs was systematically evaluated based on the results of 72 included studies and three outcomes. The results of NMA indicated that the efficacy of RDN, XYP, and YHN was better than that of ribavirin with respect to all outcome measures. With respect to total clinical effectiveness, the efficacy of SHL and TRQ was better than that of ribavirin, and the between-group difference was statistically significant. From the results of SUCRA ordering, among the three outcome indicators, RDN ranked as the best intervention, while all CHIs showed better efficacy than ribavirin. On cluster analysis, RDN was found to be the best intervention with respect to all three outcome measures. Our results highlight the efficacy of RDN in the treatment of herpangina. However, the effect of publication bias on our results cannot be ruled out; therefore, treatment decision-making in individual cases should be guided by specific situations and the experience of clinicians.

In terms of safety, 75% of the included studies monitored ADRs/ADEs. Compared with the medication monitoring of other common respiratory diseases, the RCTs included in this study were better with regard to monitoring the safety of drug use. Among the patients monitored, no significant ADRs occurred in patients treated with TRQ; therefore, its safety needs to be further confirmed by observational studies. In the reported ADRs/ADEs, except for one case of dyspnea and mild chest pain in the ribavirin group, no serious cases occurred in the other groups. The most frequently reported ADRs/ADEs of CHIs were gastrointestinal reactions, followed by rash and leukopenia. Leukopenia occurred primarily in the ribavirin group. The incidence of ADRs was most common in the YHN group, followed by the ribavirin group; the XYP group had the lowest incidence of ADRs/ADEs. Therefore, due care should be taken to avoid ADRs, especially when using YHN and ribavirin.

This is the first study that used the NMA method to evaluate the efficacy and safety of CHIs in the treatment of herpangina and ranked the results of clinical total effectiveness and the disappearing time of two main clinical symptoms. The objective was to provide evidence and recommendations for the clinical selection of drugs. However, some limitations of this study should be considered when interpreting our results: (1) The methodological quality of the included studies was not very high. Only 14 of the 72 RCTs described the correct generation of random sequences. None of the studies mentioned allocation concealment and blinding, and one study did not describe whether the two groups had comparable baseline characteristics. (2) All the included studies were published in Chinese journals; therefore, the findings may not be entirely generalizable to other settings. (3) Most of the included RCTs compared CHIs versus ribavirin, and there was a lack of a more direct comparison of two or more CHIs. (4) This meta-analysis has not been registered online.

Based on the above limitations, we make the following recommendations: (1) For future clinical RCTs, the registration of the protocol should be carried out in advance, and the study should strictly adhere to the protocol to ensure transparency of the implementation process and avoid selective reporting. (2) Future studies should use robust methods for random sequence generation (such as the use of a random number table), implement allocation concealment (e.g., with the use of opaque envelopes), and implement strict blinding to ensure the reliability of the results. (3) More studies should be conducted to evaluate the efficacy of CHIs.

## Conclusion

In conclusion, the use of CHIs was associated with improved treatment performance and could be beneficial for patients with herpangina compared to ribavirin. RDN showed the best efficacy with respect to all three outcome measures. However, more direct comparison studies of two or more CHIs are needed to further confirm the results. Future studies should include meticulous monitoring of the safety of CHIs.

## Author Contributions

JW and XD done conception and design of the network meta-analysis. XD, HW and KW performed the network meta-analysis. XD, WZ and XL assessed the quality of the network meta-analysis. XD, HW and KW analyzed study data. XD and HW wrote the paper. All authors read and approved the final version of the manuscript.

## Funding

The National Natural Science Foundation of China (grant numbers 81473547, 81673829) Young Scientists Training Program of Beijing University of Chinese Medicine.

## Conflict of Interest

The authors declare that the research was conducted in the absence of any commercial or financial relationships that could be construed as a potential conflict of interest.

## Supplementary Material

Presentation File This file contains three parts, which includes items regarding the PRISMA checklist for network meta-analysis and corresponding pages of this study, the search strategy of traditional Chinese medicine injections in PubMed database, and details about the product information of five CHIs.

## Abbreviations

CHIs, Chinese herbal injections; NMA, network meta-analysis; RDN, Reduning injection; SHL, Shuanghuanglian injection; TRQ, Tanreqing injection; XYP, Xiyanping injection; YHN, Yanhuning injection; SUCRA, Surface under the cumulative ranking curve; ADRs/ADEs, Adverse drug reactions/Adverse drug events; RCTs, Randomized controlled trials; OR, Odds ratio; MD, mean difference; IF, Inconsistency factors.
