# Supplementary material for: Comparative Efficacy of Chinese Herbal Injections for the Treatment of Herpangina: A Bayesian Network Meta-Analysis of Randomized Controlled Trials
Source: Front Pharmacol. 2020 May 15;11:693. doi: 10.3389/fphar.2020.00693 (PMC7242616; doi:10.3389/fphar.2020.00693)
Supplement: Presentation File — This file contains three parts, which includes items regarding the PRISMA checklist for network meta-analysis and corresponding pages of this study, the search strategy of traditional Chinese medicine injections in PubMed database, and details about the product information of five CHIs. [file DataSheet_1.pdf]

PRISMA 2009 Checklist

Section/Topic	#	Checklist Item	Reported on Page #
**TITLE**			
Title	1	Identify the report as a systematic review, meta-analysis, or both.	1
**ABSTRACT**			
Structured summary	2	Provide a structured summary including, as applicable: background; objectives; data sources; study eligibility criteria, participants, and interventions; study appraisal and synthesis methods; results; limitations; conclusions and implications of key findings; systematic review registration number.	1-2
**INTRODUCTION**			
Rationale	3	Describe the rationale for the review in the context of what is already known.	2
Objectives	4	Provide an explicit statement of questions being addressed with reference to participants, interventions, comparisons, outcomes, and study design (PICOS).	2
**METHODS**			
Protocol and registration	5	Indicate if a review protocol exists, if and where it can be accessed (e.g., Web address), and, if available, provide registration information including registration number.	/
Eligibility criteria	6	Specify study characteristics (e.g., PICOS, length of follow-up) and report characteristics (e.g., years considered, language, publication status) used as criteria for eligibility, giving rationale.	3
Information sources	7	Describe all information sources (e.g., databases with dates of coverage, contact with study authors to identify additional studies) in the search and date last searched.	2-3
Search	8	Present full electronic search strategy for at least one database, including any limits used, such that it could be repeated.	2-3
Study selection	9	State the process for selecting studies (i.e., screening, eligibility, included in systematic review, and, if applicable, included in the meta-analysis).	3-4
Data collection process	10	Describe method of data extraction from reports (e.g., piloted forms, independently, in duplicate) and any processes for obtaining and confirming data from investigators.	3-4
Data items	11	List and define all variables for which data were sought (e.g., PICOS, funding sources) and any assumptions and simplifications made.	3-4
Risk of bias in individual studies	12	Describe methods used for assessing risk of bias of individual studies (including specification of whether this was done at the study or outcome level), and how this information is to be used in any data synthesis.	3-4
Summary measures	13	State the principal summary measures (e.g., risk ratio, difference in means).	4
Synthesis of results	14	Describe the methods of handling data and combining results of studies, if done, including measures of consistency (e.g., I^2) for each meta-analysis.	4

PRISMA 2009 Checklist

Section/Topic	#	Checklist Item	Reported on Page #
Risk of bias across studies	15	Specify any assessment of risk of bias that may affect the cumulative evidence (e.g., publication bias, selective reporting within studies).	3-4
Additional analyses	16	Describe methods of additional analyses (e.g., sensitivity or subgroup analyses, meta-regression), if done, indicating which were pre-specified.	4
**RESULTS**			
Study selection	17	Give numbers of studies screened, assessed for eligibility, and included in the review, with reasons for exclusions at each stage, ideally with a flow diagram.	4
Study characteristics	18	For each study, present characteristics for which data were extracted (e.g., study size, PICOS, follow-up period) and provide the citations.	5
Risk of bias within studies	19	Present data on risk of bias of each study and, if available, any outcome level assessment (see item 12).	5
Results of individual studies	20	For all outcomes considered (benefits or harms), present, for each study: (a) simple summary data for each intervention group (b) effect estimates and confidence intervals, ideally with a forest plot.	5-6
Synthesis of results	21	Present the main results of the review. If meta-analyses done, include for each, confidence intervals and measures of consistency.	5-6
Risk of bias across studies	22	Present results of any assessment of risk of bias across studies (see Item 15).	5
Additional analysis	23	Give results of additional analyses, if done (e.g., sensitivity or subgroup analyses, meta-regression [see Item 16]).	5-6
**DISCUSSION**			
Summary of evidence	24	Summarize the main findings including the strength of evidence for each main outcome; consider their relevance to key groups (e.g., healthcare providers, users, and policy makers).	6-7
Limitations	25	Discuss limitations at study and outcome level (e.g., risk of bias), and at review-level (e.g., incomplete retrieval of identified research, reporting bias).	7
Conclusions	26	Provide a general interpretation of the results in the context of other evidence, and implications for future research.	7
**FUNDING**			
Funding	27	Describe sources of funding for the systematic review and other support (e.g., supply of data); role of funders for the systematic review.	8

From: Moher D, Liberati A, Tetzlaff J, Altman DG, The PRISMA Group (2009). Preferred Reporting Items for Systematic Reviews and Meta-Analyses: The PRISMA Statement. PLoS Med 6(6): e1000097. doi:10.1371/journal.pmed1000097

For more information, visit: **www.prisma-statement.org**.

Search strategy of PubMed

#1 herpangina[MeSH Terms]

#2 herpangina[Title/Abstract]

#3 herpanginas[Title/Abstract]

#4 #1 OR #2 OR #3

#5 reduning[Title/Abstract]

#6 reduning injection[Title/Abstract]

#7 shuanghuanglian[Title/Abstract]

#8 shuanghuanglian injection[Title/Abstract]

#9 tanreqing[Title/Abstract]

#10 tanreqing injection[Title/Abstract]

#11 xiyanping[Title/Abstract]

#12 xiyanping injection[Title/Abstract]

#13 yanhuning[Title/Abstract]

#14 yanhuning injection[Title/Abstract]

#15 potassium sodium dehydroandrograpolide succinate injection [Title/Abstract]

#16 #5 OR #6 OR #7 OR #8 OR #9 OR #10 OR #11 OR #12 OR #13 OR #14 OR #15

#17 randomized controlled trial[Publication Type]

#18 controlled clinical trial[Publication Type]

#19 #17 OR # 18

#20 #4 AND #16 AND #19

Search strategy of Embase

#1 herpangina[ti,ab,kw]

#2 herpanginas[ti,ab,kw]

#3 #1 OR #2

#4 reduning[ti,ab,kw]

#5 reduning injection[ti,ab,kw]

#6 shuanghuanglian[ti,ab,kw]

#7 shuanghuanglian injection[ti,ab,kw]

#8 tanreqing[ti,ab,kw]

#9 tanreqing injection[ti,ab,kw]

#10 xiyanping[ti,ab,kw]

#11 xiyanping injection[ti,ab,kw]

#12 yanhuning[ti,ab,kw]

#13 yanhuning injection[ti,ab,kw]

#14 potassium sodium dehydroandrograpolide succinate injection[ti,ab,kw]

#15 #4 OR #5 OR #6 OR #7 OR #8 OR #9 OR #10 OR #11 OR #12 OR #13 OR #14

#16 clinical[ti,ab,kw]

#17 trial[ti,ab,kw]

#18 #16 AND #17

#19 randomized controlled trial [pt]

#20 Controlled clinical trial [pt]

#21 clinical trial [pt]

#22 random*[ti,ab,kw]

#23 #18 OR #19 OR #20 OR #21 OR #22 OR #22

#24 #3 AND #15 AND #23

Search strategy of Cochrane Library

#1 herpangina[Title/Abstract/Keyword]

#2 herpanginas[Title/Abstract/Keyword]

#3 #1 OR #2

#4 reduning[Title/Abstract/Keyword]

#5 reduning injection[Title/Abstract/Keyword]

#6 shuanghuanglian[Title/Abstract/Keyword]

#7 shuanghuanglian injection[Title/Abstract/Keyword]

#8 tanreqing[Title/Abstract/Keyword]

#9 tanreqing injection[Title/Abstract/Keyword]

#10 xiyanping[Title/Abstract/Keyword]

#11 xiyanping injection[Title/Abstract/Keyword]

#12 yanhuning[Title/Abstract/Keyword]

#13 yanhuning injection[Title/Abstract/Keyword]

#14 potassium sodium dehydroandrograpolide succinate injection[Title/Abstract/Keyword]

#15 #4 OR #5 OR #6 OR #7 OR #8 OR #9 OR #10 OR #11 OR #12 OR #13 OR #14

#16 clinical[Title/Abstract/Keyword]

#17 trial[Title/Abstract/Keyword]

#18 #16 AND #17

#19 randomized controlled trial [Publication Type]

#20 Controlled clinical trial [Publication Type]

#21 clinical trial [Publication Type]

#22 random*[Title/Abstract/Keyword]

#23 #18 OR #19 OR #20 OR #21 OR #22 OR #22

#24 #3 AND #15 AND #23

Search strategy of CNKI

#1 疱疹性咽峡炎[主题]

#2 疱疹型咽峡炎[主题]

#3 咽峡疱疹[主题]

#4 咽峡疱疹溃疡[主题]

#5 传染性咽峡炎[主题]

#6 #1 OR #2 OR #3 OR #4 OR #5

#7 热毒宁[主题]

#8 热毒宁注射液[主题]

#9 热毒宁注射剂[主题]

#10 双黄连[主题]

#11 双黄连注射液[主题]

#12 双黄连注射剂[主题]

#13 痰热清[主题]

#14 痰热清注射液[主题]

#15 痰热清注射剂[主题]

#16 喜炎平[主题]

#17 喜炎平注射液[主题]

#18 喜炎平注射剂[主题]

#19 炎琥宁[主题]

#20 炎琥宁注射液[主题]

#21 炎琥宁注射剂[主题]

#22 注射用炎琥宁[主题]

#23 #7 OR #8 OR #9 OR #10 OR #11 OR #12 OR #13 OR #14 OR #15 OR #16 OR #17 OR #18 OR #19 OR #20 OR #21 OR #22

#24 随机[全文]

#25 #6 AND #23 AND #24

Search strategy of WanFang

#1 疱疹性咽峡炎[主题]

#2 疱疹型咽峡炎[主题]

#3 咽峡疱疹[主题]

#4 咽峡疱疹溃疡[主题]

#5 传染性咽峡炎[主题]

#6 #1 OR #2 OR #3 OR #4 OR #5

#7 热毒宁[主题]

#8 热毒宁注射液[主题]

#9 热毒宁注射剂[主题]

#10 双黄连[主题]

#11 双黄连注射液[主题]

#12 双黄连注射剂[主题]

#13 痰热清[主题]

#14 痰热清注射液[主题]

#15 痰热清注射剂[主题]

#16 喜炎平[主题]

#17 喜炎平注射液[主题]

#18 喜炎平注射剂[主题]

#19 炎琥宁[主题]

#20 炎琥宁注射液[主题]

#21 炎琥宁注射剂[主题]

#22 注射用炎琥宁[主题]

#23 #7 OR #8 OR #9 OR #10 OR #11 OR #12 OR #13 OR #14 OR #15 OR #16 OR #17 OR #18 OR #19 OR #20 OR #21 OR #22

#24 随机[全部]

#25 #6 AND #23 AND #24

Search strategy of VIP

#1 疱疹性咽峡炎[题名或关键词]

#2 疱疹型咽峡炎[题名或关键词]

#3 咽峡疱疹[题名或关键词]

#4 咽峡疱疹溃疡[题名或关键词]

#5 传染性咽峡炎[题名或关键词]

#6 #1 OR #2 OR #3 OR #4 OR #5

#7 热毒宁[题名或关键词]

#8 热毒宁注射液[题名或关键词]

#9 热毒宁注射剂[题名或关键词]

#10 双黄连[题名或关键词]

#11 双黄连注射液[题名或关键词]

#12 双黄连注射剂[题名或关键词]

#13 痰热清[题名或关键词]

#14 痰热清注射液[题名或关键词]

#15 痰热清注射剂[题名或关键词]

#16 喜炎平[题名或关键词]

#17 喜炎平注射液[题名或关键词]

#18 喜炎平注射剂[题名或关键词]

#19 炎琥宁[题名或关键词]

#20 炎琥宁注射液[题名或关键词]

#21 炎琥宁注射剂[题名或关键词]

#22 注射用炎琥宁[题名或关键词]

#23 #7 OR #8 OR #9 OR #10 OR #11 OR #12 OR #13 OR #14 OR #15 OR #16 OR #17 OR #18 OR #19 OR #20 OR #21 OR #22

#24 随机[任意字段]

#25 #6 AND #23 AND #24

Search strategy of SinoMed

#1 疱疹性咽峡炎[常用字段]

#2 疱疹型咽峡炎[常用字段]

#3 咽峡疱疹[常用字段]

#4 咽峡疱疹溃疡[常用字段]

#5 传染性咽峡炎[常用字段]

#6 #1 OR #2 OR #3 OR #4 OR #5

#7 热毒宁[常用字段]

#8 热毒宁注射液[常用字段]

#9 热毒宁注射剂[常用字段]

#10 双黄连[常用字段]

#11 双黄连注射液[常用字段]

#12 双黄连注射剂[常用字段]

#13 痰热清[常用字段]

#14 痰热清注射液[常用字段]

#15 痰热清注射剂[常用字段]

#16 喜炎平[常用字段]

#17 喜炎平注射液[常用字段]

#18 喜炎平注射剂[常用字段]

#19 炎琥宁[常用字段]

#20 炎琥宁注射液[常用字段]

#21 炎琥宁注射剂[常用字段]

#22 注射用炎琥宁[常用字段]

#23 #7 OR #8 OR #9 OR #10 OR #11 OR #12 OR #13 OR #14 OR #15 OR #16 OR #17 OR #18 OR #19 OR #20 OR #21 OR #22

#24 随机[全部字段]

#25 #6 AND #23 AND #24

More details about the product information of 5 CHIs

Injection name	Source	Species / Raw materials	Component ingredients to be measured	Botanical plant names	Therapeutic claims in TCM	Indications	Adverse drug reactions	Quality control reported? (Y/N)	Chemical analysis reported? (Y/N)
Reduning injection	Jiangsu Kanion Pharmaceutical Co., Ltd.	*Artemisiae Annuae Herba, Lonicerae Japonicae Flos, Gardznize Fructus*	Gardenoside, 9.0-14.0mg/mL; chlorogenic acid, 5.6-8.4mg/mL	*Artemisia annua* L., *Lonicera japonica* Thunb, *Gardenia jasminoides* J.Ellis	Clearing heat, dispelling wind, removing toxic substance	Cold due to exogenous wind-heat, marked by high fever, headache, body pain, cough, phlegm yellow; upper respiratory tract infection and acute bronchitis	Dizziness, chest congestion, xerostomia, diarrhea, nausea, vomit, pruritus, skin rash, dyspnea	Y - YBZ08202005 issued by China Food and Drug Administration	N
Tanreqing injection	Shanghai Kaibao Pharmaceutical Co., Ltd.	*Scutellariae Radix*, Bear bile powder,	Baicalin, > 0.5mg/mL; ursodeoxycholic Acid, >	*Scutellaria baicalensis* Georgi, Selenarctos	Clearing heat-toxin, dissipating phlegm	Phlegm heat obstruct lung syndrome, such as	Dizziness, nausea, vomit, pruritus, skin	Y - YBZ00912003-2007Z-2009-2012	N

		Cornu gorais , *Lonicerae Japonicae Flos, Forsythiae Fructus*	5.4mg/mL; alanine, > 1.75-3.005.4 mg/mL	thibetanus Cuvier, Capra hircus Linnaeus, *Lonicera japonica* Thunb, *Forsythia suspensa* (Thunb.) Vahl		fever, cough, expectoration , thirst, redness of tongue, and yellow fur; acute bronchitis, acute pneumonia (early)	rash, fever, chest congestion, edema, phlebitis, anaphylactic shock, dyspnea	issued by China Food and Drug Administration	
Shuanghuang lian injection	Heilongjiang Gerun Pharmaceutical Co., Ltd.; Fusen Pharmaceutical Company Limited; Heilongjiang Zbd Pharmaceutical Co., Ltd.; Shanxi Zhendong Pharmaceutic	*Scutellariae Radix*, *Lonicerae Japonicae Flos*, *Forsythiae Fructus*	Chlorogenic acid and caffeic acid, 0.21-0.32mg/mL; Baicalin, 6.5-8.7mg/mL; Forsythin, 0.12-0.18mg/mL	*Lonicera japonica* Thunb, *Forsythia suspensa* (Thunb.) Vahl *Scutellaria baicalensis* Georgi	Clearing heat-toxin, dispelling wind	Upper respiratory tract infection, pneumonia, tonsillitis, pharyngitis, etc. Which are caused by viral and bacterial infections.	decreased blood pressure, laryngeal edema, anaphylactic shock, chills, fever, pain, fatigue, nausea, vomiting, abdominal pain, diarrhea	Y - WS_3-B-2104-96-2010 issued by China Food and Drug Administration	N

	al Co., Ltd.								
Xiyanping injection	Jiangxi Qingfeng Pharmaceutical Co., Ltd.	Andrographolide sulfonate	Andrographolide sulfonate, 90.0%-110.0% of labelled amount	*Andrographis paniculata* (Burm.f.) Nees	Clearing heat-toxin, suppress cough and check dysentery	Bronchitis, tonsillitis, bacillary dysentery	Pruritus, skin rash, palpitation, diarrhea, vomit, diarrhea	Y - WS-10863 (ZD-0863) -2002-2011Z issued by China Food and Drug Administration	N
Yanhuning injection	Hainan Star Pharmaceutical Co., Ltd.; Chongqing Lummy Pharmaceutical Co.,Ltd.; Haikou Qili Pharmaceutical Co., Ltd.; Fujian Mindong Rejuvenation Pharmaceutic	Potassium Sodium Dehydroandroandrographolide Succinate	Potassium Sodium Dehydroandroandrographolide Succinate, 90.0%-110.0% of labelled amount	*Andrographis paniculata* (Burm.f.) Nees	Clearing heat-toxin, antiviral	Viral pneumonia and viral upper respiratory tract infection	Pruritus, skin rash, gastrointestinal adverse reactions (nausea, vomit, diarrhea), reduction of leukocyte, shiver, fever, dizziness, chest congestion,	Y - YBH06272009 issued by China Food and Drug Administration	N

al Co., Ltd.; Harbin Wandashan pharmaceutic al co. LTD.	palpitation.
